# The Best in the World

**Published:** 1898-07-02

**Authors:** 


### The Best in the World.

The capital speech made by Surgeon-Major-General Jameson, Director-General of the Army Medical Department, at the annual dinner of the Indian Medical Service, is of more than professional interest. On every side one hears expressions of doubt and anxiety in regard to the efficiency of our Army Medical Service; the difficulty of getting men to fill up vacancies is well known, and the hardship entailed, and the extra work thrown upon the medical officers in consequence of the smallness of their numbers, is recognised by all conversant with the position of affairs; it must then be matter of much interest to hear from the lips of one so entitled to speak with authority as is Surgeon-Major-General Jameson, that experts abroad consider our Army Medical Service, including, of course, that of the Indian Army, to be the best in the world.

The examples given and the contrasts drawn are very striking. Since the year 1860 we have had eighteen military expeditions, the medical statistics of which have been separately recorded, and by a strange coincidence the French have had exactly the same number under circumstances somewhat similar, that is to say, in tropical or semi-tropical climates. Now, comparing the mortality of these eighteen French and eighteen English expeditions, it turns out that the French loss from disease alone in relation to strength has been a little more than four times greater than the English. But more than that, we have nothing to compare with the appalling disaster of Madagascar, where the loss from disease was 30 per cent. of the entire strength. Yet in our last expedition to Ashanti (a climate just as deadly as Madagascar) the mortality was but 4 per cent. "What then was the cause of the disaster? Just this. The general officer commanding saw only one enemy—*man*—and he took no account of a far greater enemy—*disease*—and accordingly the medical and sanitary equipments, in spite of medical protests, were left at the base. The result was that seven men were killed in action and 4,189 men died from disease."

Compare again the Spanish war in Cuba and our late war in the north-western frontier of India. From the end of 1895 until March, 1897 (a period of fifteen months), Spain had an army in Cuba numbering 200,000 men. During that short time there died from yellow fever alone 13,322, and from other diseases, irrespective of killed and wounded, 40,125, while 20,000, or 10 per cent. of the force, were invalided to Spain. It is calculated that at the present moment only 70,000 out of the 200,000 men are alive. On the other hand, in the frontier war in India, the whole loss from battle and disease was only between 4 and 5 per cent., and the greatest number in hospital at the front, on the line of communication, and at the base in any one day was $11\frac{1}{2}$ per cent.

Nor are we content to be the best in the world merely in the sanitary control of our armies. In coolness, in bravery, and in steadiness under fire our army medical department may well claim to stand well to the front. A bullet struck General Woodhouse in the thigh, passed down below the knee, broke into pieces and lodged. "The Roentgen ray apparatus revealed the exact conditions, and it was determined to extract the pieces. In the middle of the operation, artificial light being used, the Afridis crawled up and suddenly blazed into the tent, sending thirteen shots through the canvas. Now that might have been a very disturbing circumstance, and apt to interfere with the perfect application of the aseptic form of surgery. And what happened? Nothing. The operation went on, and was successfully completed as if there had been no Afridi within 100 miles." Nor can we pass over the conduct of Surgeon-Captain Beyts, who, while under a hot fire, stayed to arrest the bleeding in a wounded soldier; nor that of Surgeon-Lieutenant V. Hugo, who, when the fire was too hot to permit of lights being used, and when there was no cover of any sort, struck a match at the peril of his life and examined the wound of his patient. "The match went out amid a splutter of bullets, which kicked up the dust all around, but by its uncertain light he saw the nature of the injury. The officer had already fainted from loss of blood. The doctor seized the artery, and as no other ligature was forthcoming, he remained under fire for three hours holding a man's life between his finger and thumb." Such things make us feel that the medical service of the English army is officered by men imbued with the same instincts, the same sense of honour, the same contempt for danger as actuate the combatant branches, and that, so far as it goes, it is indeed the best in the world.

